# Trajectories of muscle quantity, quality and function measurements in hospitalized older adults

**DOI:** 10.1111/ggi.14366

**Published:** 2022-03-04

**Authors:** Carly Welch, Carolyn Greig, Danielle Lewis, Zeinab Majid, Tahir Masud, Hannah Moorey, Thomas Pinkney, Benjamin Stanley, Thomas Jackson

**Affiliations:** ^1^ Medical Research Council (MRC) – Versus Arthritis Center for Musculoskeletal Aging Research University of Birmingham and University of Nottingham Birmingham UK; ^2^ Institute of Inflammation and Aging, College of Medical and Dental Sciences University of Birmingham Birmingham UK; ^3^ University Hospitals Birmingham NHS Foundation Trust Birmingham UK; ^4^ School of Sport, Exercise, and Rehabilitation Sciences University of Birmingham Birmingham UK; ^5^ Birmingham Biomedical Research Center University of Birmingham and University Hospitals Birmingham NHS Foundation Trust Birmingham UK; ^6^ Nottingham University Hospitals NHS Trust Nottingham UK; ^7^ University of Nottingham Nottingham UK; ^8^ Academic Department of Surgery University of Birmingham Birmingham UK

**Keywords:** acute sarcopenia, deconditioning, echogenicity, physical function, ultrasound

## Abstract

**Aim:**

Acute sarcopenia is defined by the development of incident sarcopenia (low muscle quantity/quality and function) within 6 months of a stressor event. However, outcome measures for clinical trials have not been validated. This study aimed to characterize changes in muscle quantity, quality, strength, and physical function during and after hospitalization.

**Methods:**

Patients aged ≥70 years admitted for elective colorectal surgery, emergency abdominal surgery or acute infections were recruited from a single university hospital. Assessments were carried out at baseline, and within 7 ± 2 days and 13 ± 1 weeks postoperatively or post‐admission.

**Results:**

A total of 79 participants (mean age 79 years, 39% female) were included. Physical function defined by the Patient‐Reported Outcome Measures Information System T‐score declined from baseline (42.3, 95% CI 40.2–44.3) to 7 days (36.6, 95% CI 34.5–38.8; *P* = 0.001), with improvement after 13 weeks (40.5, 95% CI 37.9–43.0). Changes in muscle quantity, quality and function measurements were overall heterogeneous, with few significant changes at the study population level. Change in rectus femoris echogenicity over 13 weeks correlated with changes in handgrip strength (*r* = 0.53; *P* < 0.001) and gait speed (*r* = 0.59; *P* = 0.003) over the same period.

**Conclusions:**

Patient‐Reported Outcome Measures Information System T‐score provides a sensitive measure of change in physical function in hospitalized older patients. However, changes in muscle quantity, quality and function measurements were heterogeneous, and not significant at the study population level. Further research should assess for factors that might be predictive of changes within individuals to enable stratified interventions. **Geriatr Gerontol Int 2022; 22: 311–318**.

## Introduction

Sarcopenia is defined by low muscle strength with low muscle quantity/quality, with cut‐offs two standard deviations below means of young healthy reference populations.^1^ Additional demonstration of low physical performance defines severe sarcopenia. Acute sarcopenia is a condition of acute muscle insufficiency defined by declines in muscle quantity and/or function leading to incident sarcopenia within 6 months, normally after stressor events.^1^, ^2^ However, relative declines that do not meet sarcopenia cut‐offs might also be significant.^2^ Acute sarcopenia is considered to occur commonly in older adults after hospitalization. However, changes in muscle quantity, quality and function have not been fully characterized. Characterization is vital to enable robust trial design and accurate interpretation of effectiveness. Ultrasound and bioelectrical impedance analysis are potential methods for measuring muscle quantity/quality in multiple settings.^1^, ^3^ The present study aimed to characterize changes in muscle quantity, quality and function in hospitalized older adults, and assess the relationship of changes to patient‐reported physical function at 1 week and 3 months post‐hospitalization. This was considered important in showing the relationship of change to participants' perceived function at each timepoint.

## Methods

### Study design and setting

This was a single‐site study at Queen Elizabeth Hospital Birmingham (QEHB), in Birmingham, the UK. Patients were recruited from May 2019 to April 2021. Recruitment was paused March to September 2020, and January to March 2021 due to the coronavirus disease 2019 (COVID‐19) pandemic. The protocol has been published previously.^4^ The study was prospectively registered (NCT03858192). Patients were recruited to three cohorts: elective colorectal surgery, emergency abdominal surgery and general medical patients with acute bacterial infections. Elective participants were recruited from preoperative assessment clinic, and emergency surgery and medical participants were recruited from medical and surgical wards. Baseline assessments were carried out preoperatively in the elective cohort, within 48 h of surgery in the emergency surgery cohort, and within 48 h of admission in the medical cohort. Assessments were repeated at 7 ± 2 days post‐hospitalization/surgery, and at 13 ± 1 weeks post‐hospitalization/surgery. Follow up was carried out in participants' own homes or the Inflammation Research Facility, QEHB. Due to the COVID‐19 pandemic, amendments were added in March 2020 to enable telephone follow up at 3 months and September 2020 to enable recruitment of patients with COVID‐19 to the medical cohort.^5^

### Participants

All participants were aged ≥70 years, and either provided written informed consent to participate, or a personal or professional consultee provided written consultee declaration, where they lacked capacity to do so. Prespecified exclusion criteria were life expectancy <30 days, inability to understand verbal/written English, and inability to mobilize 4 m independently 2 weeks before recruitment.

### Research procedures

Table S1 (online supplement) shows the timing of each procedure separated by cohort.

#### Ultrasound quadriceps

Ultrasound quadriceps was carried out at each visit, as previously described.^3^ Participants were positioned on a hospital bed or couch with knees extended in a natural resting position, a firm wedge placed below knee, and upper body reclined to 45^o^.^6^ The same position was established when participants were seen in their own home using recliner chairs, home couches or their own bed. Measurements were taken at the midpoint between the joint line of the knee and greater trochanter on each side. Thickness measurements of subcutaneous (SC) tissue, rectus femoris (RF) and vastus intermedius, not including the fascia, were taken in the transverse plane using B‐mode ultrasonography with a linear probe (Venue 50; GE Healthcare, Chicago, IL. USA). Three (or four if >10% variability) measures were taken on each side, and means of individual readings were used for analysis. Bilateral anterior thigh thickness (BATT) was calculated (right RF + right vastus intermedius + left RF + left vastus intermedius). BATT: SC ratio (BATT‐SCR) was calculated as BATT divided by (right SC + left SC). A single image was taken in longitudinal planes on both sides. RF grey scale analysis was carried out using Image J software (National Institutes of Health, Bethesda, MD, USA) to determine echogenicity; a marker of muscle quality.

#### Bioelectrical impedance analysis

Bioelectrical impedance analysis was carried out at each visit (Bodystat Quadscan 4000; Bodystat Limited, Douglas, Isle of Man). Participants were positioned as described for ultrasound assessment.^6^ Electrodes were applied to the right hand and foot. All available measures were extracted from the device. The phase angle was recorded as a marker of muscle quality.^7^ Skeletal muscle mass (SMM) was calculated using two equations: SMM‐Sergi and SMM‐Janssen (Table S2, Online supplement). Bioelectrical impedance analysis was not carried out in participants with cardiac devices.

#### Handgrip strength

Handgrip strength measurement was carried out at each visit using a Jamar hydraulic dynamometer. Participants were positioned in a chair (if able to sit up) or bed, with their elbow bent at 90^o^. Participants were advised to “squeeze as hard as [they] can.”^8^ Two measures were taken on each side, and the best of all four was used for analysis.

#### Physical performance

The Short Physical Performance Battery (SPPB; side‐by‐side stand, semi‐tandem, tandem stand, five chair stands and usual gait speed over 4 m) was measured at all visits in the medical cohort, in preoperative assessment clinic and 13 weeks in the elective cohort, and at 13 weeks in the emergency cohort.^9^ Usual gait speed alone was measured at 7 days in surgical cohorts.

#### Patient‐Reported Outcomes Measurement Information System (physical function)

The Patient‐Reported Outcomes Measurements Information System (PROMIS) item bank V2.0 Physical Function Short Form 10b questionnaire was administered at baseline, 7 days, and at 13 weeks.^10^ In emergency surgery and medical cohorts, participants were asked to answer according to perceived physical function 2 weeks before admission. Raw scores were entered into the HealthMeasures Scoring Service, powered by Assessment Center to derive T‐scores.

#### Sarcopenia diagnosis

Sarcopenia was defined according to previously defined cut‐offs as reduced handgrip strength (<27 kg in men, <16 kg in women),^1^ and reduced BATT (<5.44 cm in men, <3.85 cm in women)^3^ and/or reduced SMM‐Sergi (<20 kg in men, <15 kg in women).^1^ We calculated the prevalence of sarcopenia at baseline and the prevalence of acute sarcopenia at 7 days. We also further calculated the prevalence of participants who experienced negative changes in muscle quantity, strength or performance of ≥10%, but who did not meet criteria for sarcopenia at 7 days.

### Statistical analysis

Statistical analyses were carried out using IBM spss Statistics 26 (IBM Corporation, Armonk, NY, USA). One‐way analysis of variance (anova), χ^2^‐tests, Kruskal–Wallis tests and Mann–Whitney *U*‐tests were used to assess for significance of differences in baseline characteristics, and baseline muscle and physical function measurements between cohorts. The study was originally powered (80% power, alpha 0.05) to assess within‐group differences in PROMIS scores from baseline to 13 week follow up (56 participants in each cohort; 45 to follow up with 25% dropout rate). Due to the study being paused, the recruitment target was revised for differences across groups (45 to follow up across groups). To enable comparisons across groups, main analyses were carried out across three visits for all groups, to assess changes to 7 days and 13 weeks compared with baseline. Preoperative assessments were used in the elective cohort, and postoperative assessments were used in the emergency surgery cohort (i.e. at recruitment for most participants). Linear mixed models (normally distributed variables) and generalized linear mixed models (non‐normal distributed variables) were used to assess for the significance of differences in muscle and physical function variables between visits, including an interaction term for visit and group. Mixed models are considered robust to effects of missing values. Estimated marginal means were derived from models. Analyses were separated by sex for variables with sex‐specific sarcopenia cut‐offs. Secondary analyses for within cohort differences across all visits were carried out using linear mixed models and generalized linear mixed models . Change scores from baseline to 7 days and 13 weeks were calculated for all muscle quantity, quality and physical function measurements. Correlation matrices (Pearson and Spearman) of change scores were generated using GraphPad Prism 9. Multivariate analyses were planned to assess if changes in muscle quantity, quality and function measurements within 7 days were predictive of change in PROMIS score at 13 weeks. However, on evaluation of correlation matrices, multivariate analyses were not indicated.

## Results

### Participant characteristics at baseline

Feasibility analyses including screening, recruitment and dropouts have been published separately.^11^ A total of 81 participants were recruited. One participant was excluded from the emergency surgery cohort (elective admission recruited in error). One further emergency surgery participant was excluded from baseline and main analyses, as only preoperative measurements were carried out (did not undergo surgery). Figure S1 (online supplement) shows dropouts within each cohort. Table 1 shows baseline characteristics for participants, separated by cohort. Participants in the medical cohort were older (mean age 82.1 *vs* 76.4 in elective surgery cohort, 75.2 in emergency surgery cohort; *P* < 0.001), at greater risk of being malnourished and more frail than surgical cohorts. There were no significant differences in muscle quantity or quality between cohorts. However, medical participants had lower physical function at baseline in terms of both physical performance (median 0.33 *vs* 0.76 m/s in elective surgery; *P* < 0.001) and PROMIS T‐scores (36.8 *vs* 47.7 in elective surgery; *P* < 0.001).

**Table 1:** Baseline characteristics, and muscle and physical function assessments for participants separated by patient cohort

	Overall (*n* = 79)	Elective surgery (*n* = 24)	Emergency surgery (*n* = 14)	Medical (*n* = 41)	*P*‐value	
Baseline characteristics						
Age, mean (SD)	79.1 (6.6)	76.4 (5.3)	75.2 (4.2)	82.1 (6.7)	<0.001^a^	
Sex, females % (*n*)	39.2 (31)	50.0 (12)	35.7 (5)	34.1 (14)	0.431^b^	
Ethnicity, % (*n*)						
White British	93.7 (74)	95.8 (23)	100 (14)	90.2 (37)	0.742^b^	
White Irish	2.5 (2)	0 (0)	0 (0)	4.9 (2)		
Indian	2.5 (2)	4.2 (1)	0 (0)	2.4 (1)		
Arab	1.3 (1)	0 (0)	0 (0)	2.4 (1)		
Body mass index (kg/m^2^), mean (SD)	26.5 (6.5)	26.4 (4.3)	24.3 (4.3)	27.4 (8.0)	0.303^a^	
Nutritional status, % (*n*)						
Normal	41.8 (33)	75.0 (18)	35.7 (0)	24.4 (10)	0.001^b^	
At risk	50.6 (40)	25.0 (6)	64.3 (9)	61.0 (25)		
Malnourished	7.6 (6)	0 (0)	0 (0)	14.6 (6)		
Frailty index, mean (SD)	0.27 (0.11)	0.20 (0.09)	0.25 (0.14)	0.32 (0.09)	<0.001^a^	
Clinical Frailty Scale, median (IQR)	4 (3–5)	3 (3–4)	3.5 (2.75–4)	5 (4–5)	<0.001^c^	
Baseline muscle and physical function assessments						
BATT (cm), mean (SD)					
Male	4.49 (1.21)	4.67 (1.07)	4.91 (1.11)	4.24 (1.29)	0.318^a^
Female	3.69 (1.14)	3.60 (1.15)	3.75 (0.70)	3.73 (1.28)	0.953^a^
BATT‐SCR, median (IQR)					
Male	3.57 (2.32–5.09)	4.16 (2.33–5.40)	3.98 (2.77–5.87)	3.20 (1.95–4.24)	0.293^c^
Female	1.59 (1.15–2.65)	1.36 (1.17–3.10)	2.32 (0.95–2.73)	1.71 (1.12–2.80)	0.948^c^
Echogenicity, mean (SD)					
Male	63.3 (13.0)	58.3 (13.9)	65.4 (13.6)	65.8 (11.8)	0.272^a^
Female	70.0 (13.6)	72.4 (16.5)	63.5 (4.8)	70.2 (13.1)	0.485^a^
SMM‐Janssen (kg) – median (IQR)					
Male	24.7 (21.0–28.2)	22.6 (20.9–30.6)	25.8 (24.0–29.6)	24.7 (18.0–27.1)	0.702^c^
Female	16.9 (15.8–20.7)	17.9 (14.7–25.6)	16.4 (16.0–18.8)	16.7 (15.2–20.8)	0.274^c^
SMM‐Sergi (kg), mean (SD)					
Male	21.5 (4.7)	21.4 (4.7)	21.9 (2.5)	21.3 (5.7)	0.946^a^
Female	16.5 (4.9)	17.6 (5.1)	14.0 (1.8)	16.4 (5.2)	0.471^a^
Phase angle (^o^), median (IQR)					
Male	4.60 (3.90–5.30)	4.70 (4.60–5.80)	4.20 (3.65–4.65)	4.40 (3.80–5.30)	0.124^c^
Female	4.80 (4.00–5.50)	5.30 (4.80–5.50)	4.30 (3.93–4.75)	4.25 (3.65–5.50)	0.072^c^
Handgrip strength (kg), mean (SD)					
Male	23.1 (9.3)	26.6 (10.7)	25.3 (9.1)	20.4 (8.1)	0.123^a^
Female	14.8 (8.1)	19.1 (7.9)	12.2 (5.4)	12.7 (7.9)	0.074^a^
Gait speed (m/s), median (IQR)	0.58 (0.19–0.76)	0.76 (0.67–0.89)	NA	0.33 (0–0.55)	<0.001^d^	
SPPB, median (IQR)	6.50 (1.00–9.00)	9.00 (8.00–10.75)	NA	1.50 (0–5.00)	<0.001^d^	
PROMIS T‐score, mean (SD)	41.1 (9.5)	47.7 (9.5)	42.3 (10.0) *P* = 0.145	36.8 (9.0) *P* < 0.001	<0.001^a^	

^a^ One‐way anova.

^b^ χ^2^‐test.

^c^ Kruskal–Wallis test.

^d^ Mann–Whitney U‐test.

BATT, bilateral anterior thigh thickness; BATT‐SCR, bilateral anterior thigh thickness : subcutaneous tissue ratio; PROMIS, patient‐reported outcomes measurement information system; SMM‐Janssen, skeletal muscle mass (Janssen equation); SMM‐Sergi, skeletal muscle mass (Sergi equation); SPPB, short physical performance battery.

### Dynamic changes in muscle quantity, quality and function measurements

**Figure 1:**
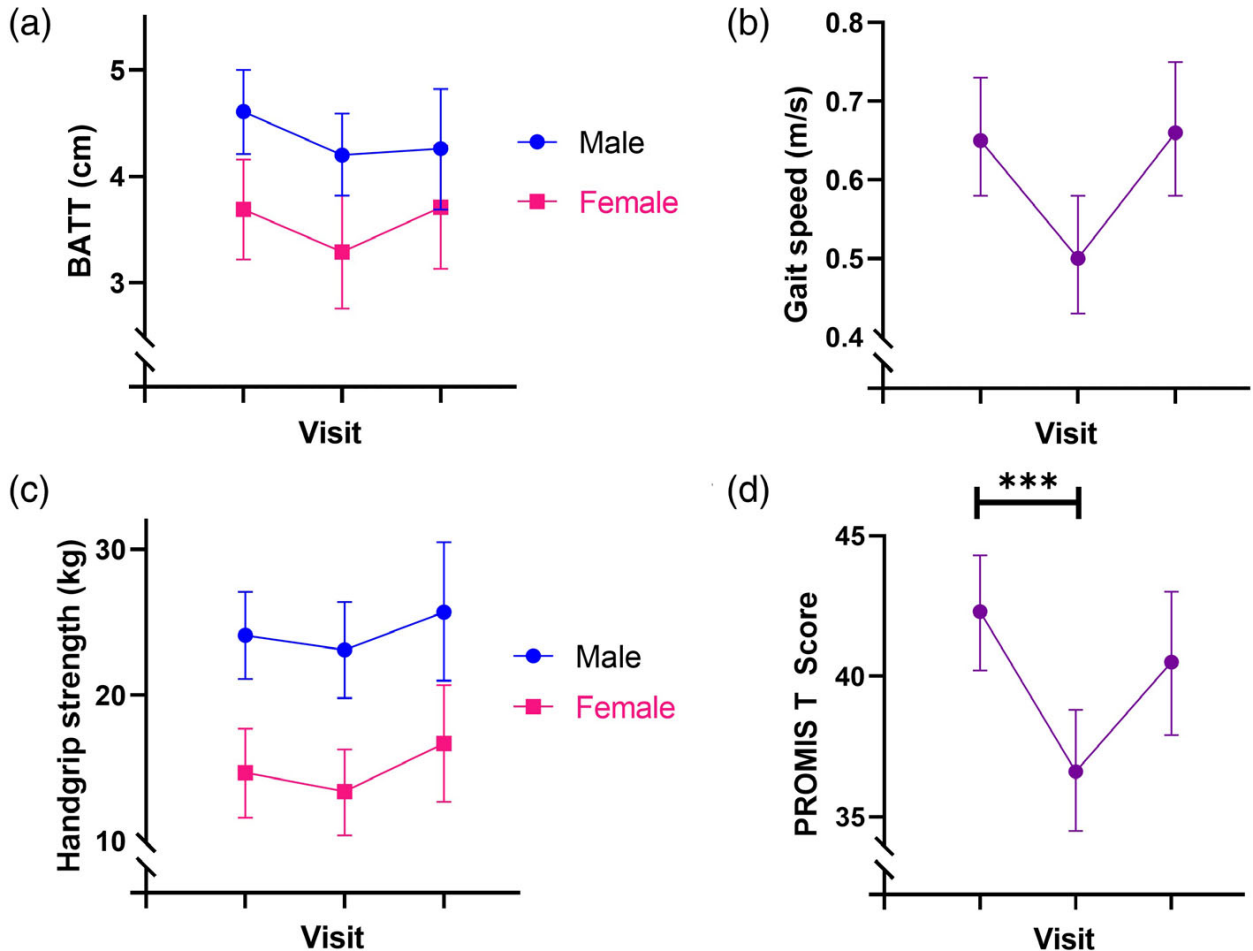
Changes in estimated marginal means of muscle quantity and function measurements between visits. Error bars are 95% confidence intervals. BATT, bilateral anterior thigh thickness.

Table 2 shows estimated marginal means and 95% confidence intervals for measurements across each visit across groups. There was a general trend across all measures toward reduction at 7 days compared with baseline. However, most changes were not statistically significant. PROMIS T‐scores significantly declined from baseline to 7 days postoperative/post‐admission. However, scores recovered toward baseline at 13 weeks, with a similar pattern seen with gait speed (Fig. 1). Figure S3 shows the prevalence of acute sarcopenia at 7 days, as well as the percentage of participants who experienced negative changes in muscle quantity, strength or physical performance, but who did not meet criteria for sarcopenia at 7 days. Of those participants who did not meet criteria for sarcopenia at baseline, just 22.2% did not experience negative changes of ≥10% or meet the criteria for acute sarcopenia.

**Table 2:** Estimated marginal means derived from linear mixed models and generalized linear mixed models

	Baseline	7 ± 2 days post‐admission/surgery	13 ± 1 weeks post‐admission/surgery	*P*‐value		
				Visit	Group	Visit × group
BATT (cm)						
Male	4.61 (4.21–5.00)	4.20 (3.82–4.59)	4.26 (3.69–4.82)	0.310^a^	0.012^a^	0.543^a^
Female	3.69 (3.22 – 4.16)	3.29 (2.76–3.82)	3.71 (3.13–4.29)	0.430^a^	0.533^a^	0.827^a^
BATT‐SCR						
Male	4.02 (3.38–4.79)	3.64 (3.03–4.37)	3.96 (2.83–5.54)	0.714^b^	0.033^b^	0.934^b^
Female	1.97 (1.58–2.46)	1.74 (1.39–2.17)	2.03 (1.53–2.69)	0.592^b^	0.764^b^	0.939^b^
Echogenicity						
Male	63.2 (58.7–67.8)	64.1 (59.0–69.2)	61.0 (53.6–68.4)	0.769^a^	0.969^a^	0.561^a^
Female	68.9 (63.5 – 74.4)	71.9 (64.6–79.1)	67.8 (59.7–75.9)	0.707^a^	0.916^a^	0.669^a^
SMM‐Janssen (kg)						
Male	25.0 (22.7–27.4)	24.1 (21.5–27.0)	20.6 (18.7–22.7)	0.013^b^	0.004^b^	0.068^b^
Female	18.5 (16.1–21.3)	17.0 (14.6–19.9)	18.3 (15.3–21.8)	0.694^b^	0.274^b^	0.023^b^
SMM‐Sergi (kg)						
Male	21.6 (19.9–23.2)	21.0 (19.2–22.8)	20.6 (17.9–23.2)	0.777^a^	0.590^a^	0.981^a^
Female	16.0 (13.9 – 18.1)	15.3 (13.1–17.5)	15.5 (12.4–18.5)	0.878^a^	0.037^a^	0.808^a^
Phase angle (^o^)						
Male	5.87 (4.86–7.10)	4.47 (4.19–4.77)	5.87 (4.86–7.10)	0.026^b^	0.485^b^	0.082^b^
Female	4.95 (4.23–5.79)	4.82 (4.15–5.59)	5.32 (4.63–6.11)	0.556^b^	0.095^b^	0.369^b^
Handgrip (kg)						
Male	24.1 (21.1–27.1)	23.1 (19.8–26.4)	25.7 (21.0–30.5)	0.648^a^	0.022^a^	0.549^a^
Female	14.7 (11.6 – 17.7)	13.4 (10.4–16.3)	16.7 (12.7–20.7)	0.384^a^	0.002^a^	0.870^a^
Gait speed (m/s)	0.65 (0.58–0.73)	0.50 (0.43–0.58)	0.66 (0.58–0.75)	0.004^b^	<0.001^b^	0.426^b^
SPPB	6.19 (5.24–7.32)	4.25 (3.01–5.85)	6.99 (5.97–8.19)	0.904^b^	<0.001^b^	0.290^b^
PROMIS T‐score	42.3 (40.2–44.3)	36.6 (34.5–38.8)	40.5 (37.9–43.0)	0.001^a^	<0.001^a^	0.302^a^

^a^ Linear mixed models.

^b^ Generalized linear mixed model. The 95% confidence intervals are shown in parentheses.

BATT, bilateral anterior thigh thickness; BATT‐SCR, bilateral anterior thigh thickness : subcutaneous tissue ratio; PROMIS, patient‐reported outcomes measurement information system; SMM‐Janssen, skeletal muscle mass (Janssen equation); SMM‐Sergi, skeletal muscle mass (Sergi equation); SPPB, short physical performance battery.

### Correlations of individual change scores in PROMIS with other measurements

**Figure 2:**
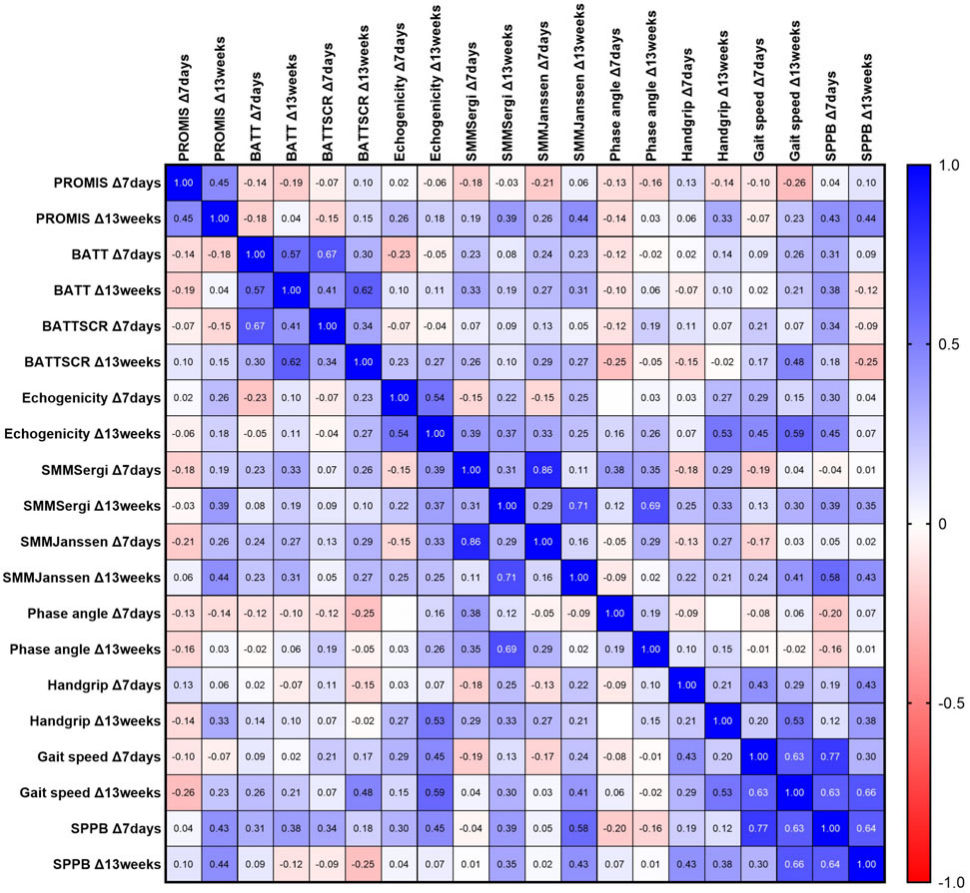
Correlation matrix derived from Pearson correlations. BATT, bilateral anterior thigh thickness; BATT‐SCR, bilateral anterior thigh thickness : subcutaneous tissue ratio; PROMIS, Patient‐Reported Outcome Measures Information System, Physical Function; SMM‐Sergi, skeletal muscle mass (Sergi equation); SMM‐Janssen, skeletal muscle mass (Janssen equation); SPPB, Short Physical Performance Battery.

**Figure 3:**
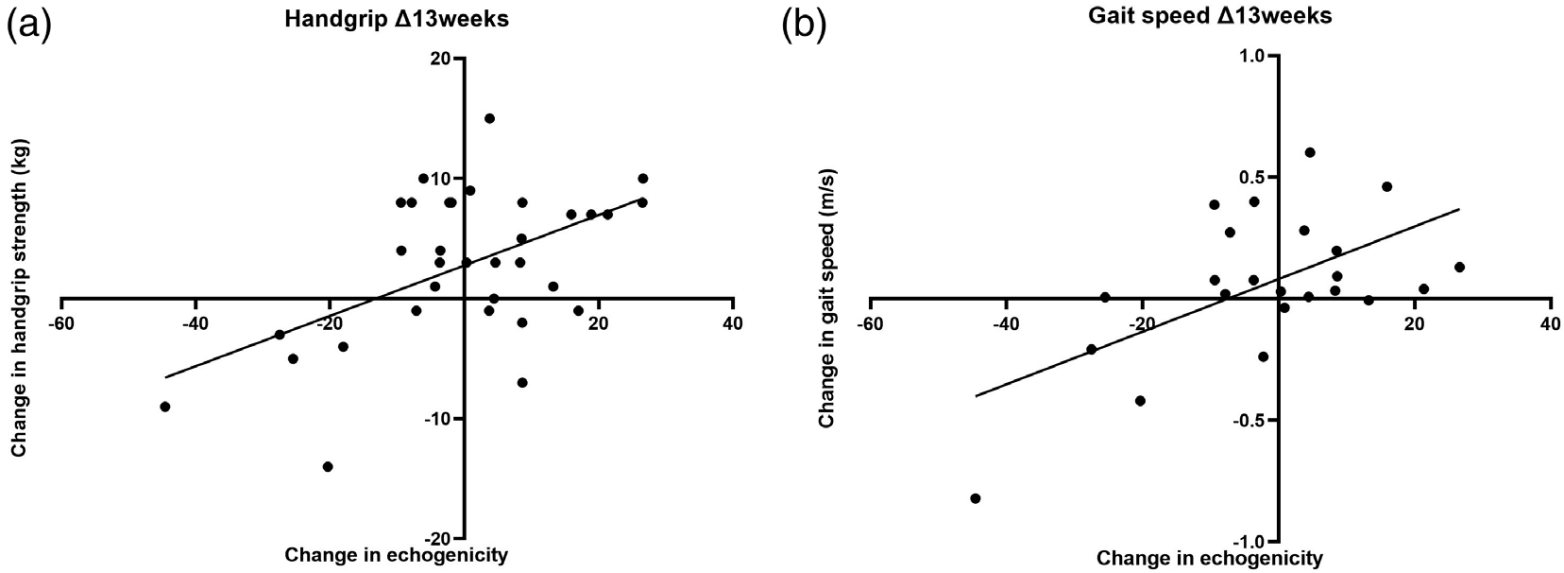
Association of echogenicity between handgrip strength and gait speed. Trend lines are derived from simple linear regression.

Figure 2 shows the Pearson correlation matrix for change scores of muscle quantity, quality and function measurements at 7 days and 13 weeks. Spearman correlations produced similar results (Figure S2, online supplement). There were no significant correlations with change in PROMIS T‐score at 7 days. There were moderate correlations between change in PROMIS T‐score at 13 weeks, and changes in PROMIS T‐score and SPPB at 7 days, and changes in SMM‐Janssen and SPPB at 13 weeks. There were also moderate correlations between the change in echogenicity at 13 weeks and change in gait speed and SPPB at 7 days, and change in gait speed and handgrip strength at 13 weeks (Fig. 3).

### Discussion

Baseline measurements of muscle quantity and quality did not significantly differ between groups. This is despite medical participants showing greater levels of frailty, being more likely to be malnourished, and having lower patient‐reported physical function and physical performance scores. Previous studies have shown lower prevalence of frailty amongst surgical compared to medical patients. However, previous studies evaluating muscle quantity and function in hospitalized older adults have focused on single patient groups,^12^ or analyzed changes and differences overall combining different specialty populations.^13^, ^14^

Overall, minimal changes in muscle quantity, quality or function were shown at the study population level. This is consistent with previous studies that have not shown significant change in handgrip strength in acutely admitted older adults during hospitalization,^15^, ^16^ or at 3 months post‐hospitalization.^13^ A previous systematic review showed declines in handgrip strength in electively admitted older adults, but not in acutely admitted patients.^16^ Conversely, muscle quantity has been shown to decline at 3 months post‐hospitalization,^13^ but not during hospitalization,^14^ and physical performance has actually been shown to improve in other studies.^13^, ^15^ This shows complexities in measuring dynamic changes in muscle quantity, quality and function in heterogeneous populations. Importantly, although changes were not shown at the study population level, some individuals experienced significant negative changes. Previous interventional trials have often examined for effect sizes at study population levels.^17^ However, unless interventions are targeted toward individuals most likely to experience negative changes, it might not be possible to show effectiveness.

Gait speed declined significantly at 7 days post‐admission/postoperatively. However, this might have been affected by factors, such as pain, and restraint from intravenous fluids and catheters. This shows the need for caution when carrying out studies measuring physical performance during hospitalization, where assessment at a single timepoint might provide an incomplete clinical picture. In this hospital, enhanced recovery after surgery, including early mobilization, is part of the standard care for patients undergoing elective colorectal surgery.^18^

Knee extension strength was not measured as part of the present study, but has been shown to decline during hospitalization.^15^ Knee extension strength has been shown to be more sensitive to change in resistance exercise trials in frail older adults than handgrip strength.^19^ It is likely that different muscles might respond differently to hospital‐associated inactivity/disuse. Hospitalization might be associated with prolonged periods of bedrest, with limited lower limb use, but continuous upper limb use. Lower limb anti‐gravity muscles might be more susceptible to declines in function than upper limb muscles.

Where changes did occur, these were infrequently correlated. This suggests there might be multiple mechanisms affecting changes. This is potentially very important to consider, as all changes might be individually significant. Identifying mechanistic pathways for individual changes is imperative to ensure that most suitable outcomes are included within trials that seek to target specific pathways. It is important to consider that many participants experienced negative declines of ≥10% in individual domains, but did not meet the criteria for sarcopenia; some participants experienced declines in all domains without meeting criteria for sarcopenia. This shows the importance of considering dynamic changes, as these relative declines are likely to be individually important.

Notably, changes in PROMIS scores were shown acutely during hospitalization. This confirms that the PROMIS physical function score itself is sensitive to change in an older hospitalized population, and might be an appropriate outcome measure in large‐scale clinical studies. However, PROMIS scores are reliant on participants' own perceptions. Although this can be considered a strength, the lack of objectivity means that scores might not be appropriate outcomes for early‐stage efficacy trials aimed at showing mechanisms underlying interventions. PROMIS provides a measure of participants' own perceptions of what they are able to do, rather than an objective assessment of what they can do. Responses might, therefore, vary according to mood, cognition, cultural background or outlook on life.^10^ Responses might also differ when obtained from proxies.^20^

Changes in PROMIS scores did not clearly correlate with changes in other measurements. This suggests that the PROMIS score might be affected by multidimensional factors, and not just intrinsic muscle factors. Hospitalization might be associated with symptoms of fatigue,^21^ low mood,^22^ cognitive impairment,^21^ physical restraints from indwelling catheters and lines,^23^ as well as disease‐specific symptoms, such as nausea,^24^ pain^21^ and breathlessness.^25^ All of these factors might lead to impairments in physical function that are not intrinsically muscle‐related. Understanding these factors is imperative to considering how interventions are targeted to prevent negative changes in physical function.

Change in RF echogenicity, but not muscle quantity measures, correlated with change in function measures (handgrip strength and gait speed) over 13 weeks. Echogenicity is considered to relate to intramuscular adipose deposition, and provides a measure of muscle quality. This suggests that muscle quality might be more important for maintenance of muscle function than muscle quantity. This is consistent with previous cross‐sectional research in stable older adults, which showed that RF echogenicity correlated with handgrip strength and gait speed.^3^ However, change in echogenicity was not associated with change in function over 7 days. This might relate to effects of fluid shifts on echogenicity; increased edema and extracellular fluid (e.g. postoperatively) might lead to reductions in echogenicity, as water will appear more black on ultrasound imaging.^26^ Fatigue and compliance with handgrip strength and physical performance assessment in the acute setting might impact on these measures. Alternatively, this might suggest that muscle quality is more relevant in development of chronic sarcopenia, with development over longer time periods.

Importantly, associations do not necessarily imply causation, or direction or causality. Low muscle quality (low echogenicity) might develop as a consequence of reduced muscle activity (presenting as low handgrip strength/gait speed), muscle function might be reduced directly by reduced muscle quality or there might be intermediary factors affecting all measures. Considering trends shown in Figure 3, it should be noted that, although some individuals experienced reductions in muscle quality and function, other individuals experienced improvements. Understanding differences between these groups is imperative toward deciphering mechanisms, and carefully targeting and stratifying interventions.

Large cohort studies to fully characterize changes during and after hospitalization are encouraged, with implementation of techniques, such as latent class association, to understand what is different about those who experience improvements in muscle quantity, quality and function, compared with those who experience declines. Individual follow up to understand how changes impact on much longer‐term outcomes would also be beneficial. Such studies could potentially be embedded into longitudinal studies to enable collection of pre‐insult measurements, even in unscheduled admissions.

Mechanistic studies are warranted to understand pathways associated with phenotypic changes. Ideally, such studies should incorporate serial muscle biopsies to enable enhanced understanding that could lead to the development of novel interventions. At the same time, interventional studies should not be delayed, and studies might need to have both applied health and translational remits. Early stage clinical trials might need to pragmatically include multiple outcomes to assess mechanisms and efficacy.

We recognize that there are limitations of the present study. First, due to the need to pause recruitment during the COVID‐19 pandemic, this study was underpowered compared with the original planned sample size. The study was powered sufficiently to assess differences across groups, but we cannot rule out the possibility that more significant differences might have been identified within groups in a larger sample size. Second, participants were recruited from a single site, and results might not be broadly representative elsewhere; importantly, most participants were white British. Additionally, recruitment and follow up of participants was led by a single researcher who also carried out the main statistical analysis, and was not blinded to analysis of results. Finally, we acknowledge that effects of missing values and participant dropout are unknown.

Older adults showed acute declines in their own perceived physical function after hospitalization. However, this did not clearly relate to changes in muscle quantity or quality. Changes in muscle echogenicity within 13 weeks of hospitalization were associated with changes in handgrip strength and gait speed. Further research should assess for class associations to enable stratification towards targeted interventions.

## Ethical approval

This research was sponsored by and reviewed by the University of Birmingham research governance team. Ethical approval was obtained from Wales Research Ethics Committee 4 (19/WA/0036), the Health Research Authority, and the University Hospitals Birmingham NHS Trust Research and Development department. Written informed consent was obtained from all participants who were considered to have capacity to consent for themselves. Written personal or professional consultee declaration was obtained if the participant was considered to lack the capacity to consent to participation. The use of both informed consent and consultee declaration was approved by the ethics committee.

## Conflict of interest

The authors declare no conflict of interest.

## Supporting information


**Table S1**: Research procedures carried out at each timepoint and included within analysis separated by cohort. Colored squares designate timepoints where these were performed. Timepoints where these were not performed are shown in black.
**Table S2**: Equations used in calculation of skeletal muscle mass using bioelectrical impedance analysis. In both equations: height in cm; sex 1 = male, 0 = female; weight in kg; resistance in Ω; reactance in Ω.
**Figure S1**: Recruitment and dropouts of participants across visits for each patient cohort.
**Figure S2**: Correlation matrix using Spearman correlations.
**Figure S3**: Percentage of participants meeting criteria for acute sarcopenia at 7 days, or negative changes ≥10% in those who did not meet criteria for sarcopenia.

## Data Availability

The anonymised dataset is available from the corresponding author upon reasonable request.
