# Supplementary material for: Trajectories of muscle quantity, quality and function measurements in hospitalized older adults
Source: Geriatr Gerontol Int. 2022 Mar 4;22(4):311–8. doi: 10.1111/ggi.14366 (PMC9313889; doi:10.1111/ggi.14366)
Supplement: Supplementary file 1 — Table S1: Research procedures carried out at each timepoint and included within analysis separated by cohort. Colored squares designate timepoints where these were performed. Timepoints where these were not performed are shown in black. Table S2: Equations used in calculation of skeletal muscle mass using bioelectrical impedance analysis. In both equations: height in cm; sex 1 = male, 0 = female; weight in kg; resistance in Ω; reactance in Ω. Figure S1: Recruitment and dropouts of participants across visits for each patient cohort. Figure S2: Correlation matrix using Spearman correlations. Figure S3: Percentage of participants meeting criteria for acute sarcopenia at 7 days, or negative changes ≥10% in those who did not meet criteria for sarcopenia. [file GGI-22-311-s001.docx]

# Trajectories of muscle quantity, quality, and function measurements in hospitalised older adults

## Supplementary material

Table S1: Research procedures performed at each timepoint and included within analysis separated by cohort. Coloured squares designate timepoints where these were performed. Timepoints where these were not performed are shown in black.

|  | Elective surgery | | | Emergency surgery | | | Medical | | |
| --- | --- | --- | --- | --- | --- | --- | --- | --- | --- |
|  | Pre-operative | 7 (+/-2) days post surgery | 13 (+/-1) weeks post surgery | Within 48hr of surgery | 7 (+/-2) days post surgery | 13 (+/-1) weeks post surgery | Within 48hr of admission | 7 (+/-2) days post admission | 13 (+/-1) weeks post admission |
| Ultrasound quadriceps |  |  |  |  |  |  |  |  |  |
| Bioelectrical impedance analysis |  |  |  |  |  |  |  |  |  |
| Handgrip strength |  |  |  |  |  |  |  |  |  |
| SPPB |  |  |  |  |  |  |  |  |  |
| Gait speed |  |  |  |  |  |  |  |  |  |
| PROMIS Physical Function |  |  |  |  |  |  |  |  |  |

Table S2: Equations used in calculation of Skeletal Muscle Mass (SMM) using bioelectrical impedance analysis. In both equations: Height in cm; Sex 1=male, 0=female; Weight in kg; Resistance in Ω; Reactance in Ω

| Skeletal Muscle Parameter | Equation |
| --- | --- |
| SMM-Sergi | = -3.964 + [0.227 × (height^2^/resistance)] + (0.095 × weight) + (1.384 × Sex) + (0.064 × reactance) |
| SMM-Janssen | = [(height^2^/resistance) × 0.401] + (Sex × 0.3825) + (Age × -0.071) + 5.102 |
| Phase angle | = arctan(reactance/resistance)  *“arctan" is the inverse trigonomic function (arc tangent) of the tangent function* |


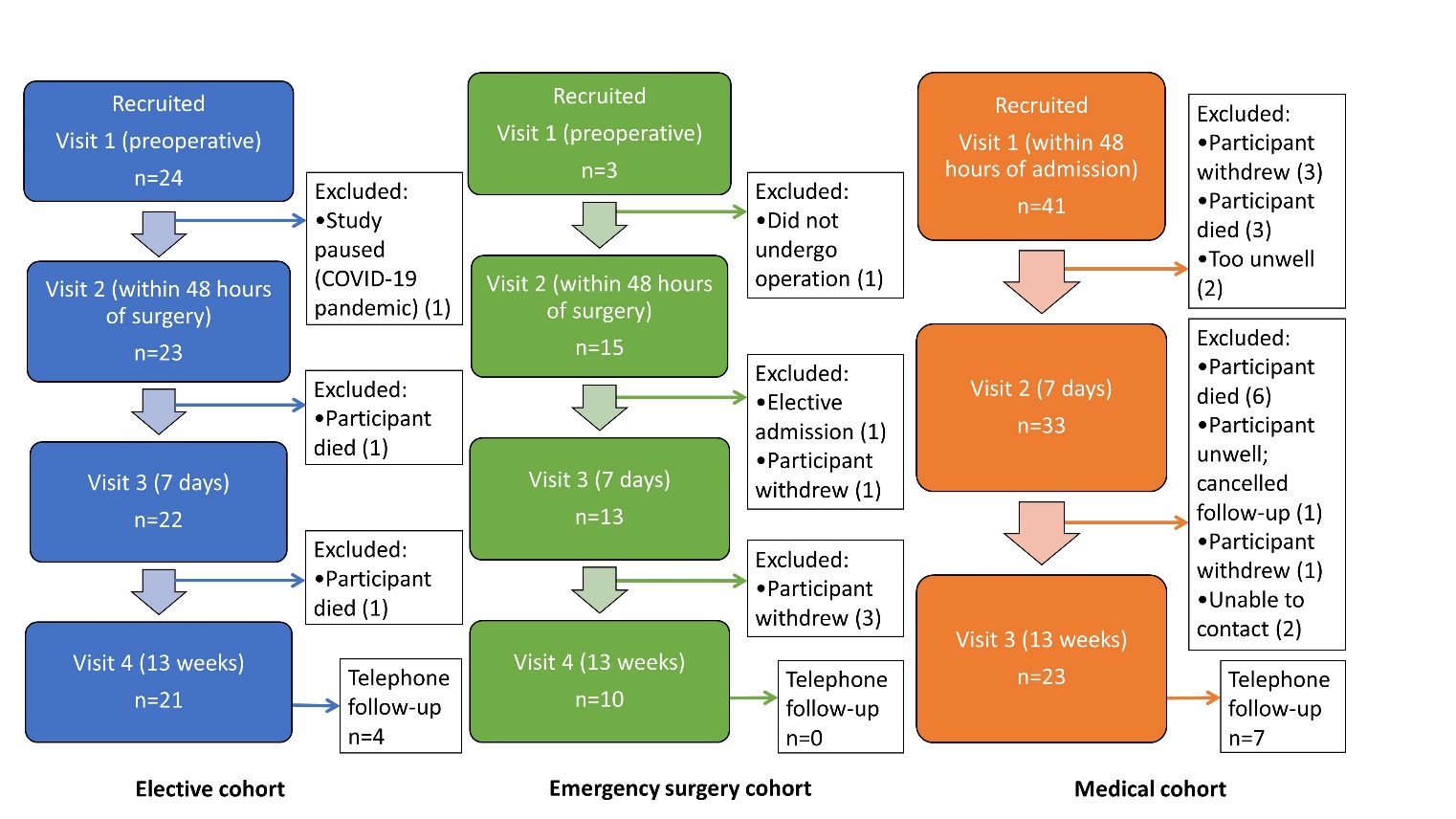


Figure S1 – Recruitment and drop outs of participants across visits for each patient cohort.

In the emergency surgery cohort, 13/16 participants were recruited post-operatively. Of the 3/16 participants recruited preoperatively, one was excluded as they did not undergo an operation. The data presented in the manuscript relate to postoperative assessments from visit 2 onwards – data collected preoperatively are not presented for the two participants for whom this was available, and were not excluded from analysis.

Figure S2 – Correlation matrix using Spearman correlations

Figure S3 – Percentage of participants meeting criteria for acute sarcopenia at 7 days, or negative changes greater than or equal to 10% in those who did not meet criteria for sarcopenia
